# The LMSz method - an automatable scalable approach to constructing gene-specific growth charts in rare disorders

**DOI:** 10.1038/s41431-025-01947-1

**Published:** 2025-10-13

**Authors:** Karen J. Low, Julia Foreman, Rachel J. Hobson, Hannah Kwuo, Elena Martinez-Cayuelas, Berta Almoguera, Purin Marin-Reina, Stefano G. Caraffi, Livia Garavelli, Emily Woods, Meena Balasubramanian, Allan Bayat, Charlotte W. Ockeloen, Caroline M. Wright, Helen V. Firth, Tim J. Cole

**Affiliations:** 1 Centre for Academic Child Health, Bristol Medical School, University of Bristol, Bristol, UK; 2 Department of Clinical Genetics, UHBW NHS Trust, Bristol, UK; 3 European Molecular Biology Laboratory, European Bioinformatics Institute, Wellcome Genome Campus, Hinxton, Cambridge UK; 4 DDD team, Wellcome Sanger Institute, Hinxton, Cambridge UK; 5 Department of Pediatrics, Hospital Universitario Fundación Jiménez Díaz, Madrid, Spain; 6 Department of Genetics and Genomics, Fundacion Jimenez Diaz University Hospital, Health Research Institute-Fundacion Jimenez Diaz, Universidad Autonoma de Madrid (IIS-FJD UAM), Madrid, Spain; 7 Center for Biomedical Network Research on Rare Diseases (CIBERER), Madrid, Spain; 8 Dysmorphology and Neonatology Unit. Hospital Universitari i Politècnic La Fe, Valencia, Spain; 9 Medical Genetics Unit, Azienda USL-IRCCS di Reggio Emilia, Reggio Emilia, Italy; 10 Division of Clinical Medicine, University of Sheffield, Sheffield, UK; 11 Department of Clinical Genetics, Sheffield Children’s Hospital, Sheffield, UK; 12 Department of Regional Health Research, University of Southern Denmark, Odense, Denmark; 13 Department of Epilepsy Genetics and Personalized Medicine, Danish Epilepsy Center, Dianalund, Denmark; 14 Department of Drug Design and Pharmacology, University of Copenhagen, Copenhagen, Denmark; 15 Department of Human Genetics, Radboud University Medical Center, Nijmegen, The Netherlands; 16 Department of Human Nutrition, School of Medicine, Dentistry and Nursing, University of Glasgow, Glasgow, UK; 17 Department of Clinical Genetics, Addenbrookes Hospital, Cambridge, UK; 18 UCL Great Ormond Street Institute of Child Health, London, UK

**Keywords:** Growth disorders, Genetic testing

## Abstract

Children with monogenic neurodevelopmental disorders often grow abnormally. Gene-specific growth charts would be useful but require large samples to construct them using the conventional LMS method. We transformed anthropometry to British 1990 reference z-scores for 328 UK and 264 international individuals with *ANKRD11, ARID1B, ASXL3, DDX3X, KMT2A*, or *SATB2-*related disorders, and modelled mean and standard deviation (SD) of the z-scores as gene-specific linear age trends adjusted for sex. Assuming the same skewness in the reference and rare disease distributions, we then back-transformed the mean ±2 SD lines to give gene-specific median, 2nd, and 98th centiles. The resulting z-score charts look plausible on several counts. Only *KMT2A* shows a (rising) age trend in median height, while BMI and weight increase for several genes, possibly reflecting population trends. Apart from *SATB2* and *DDX3X*, the gene-specific medians are all below the reference (range 0.1th centile for height *KMT2A* to 36th centile for BMI *ANKRD11*). Median OFC z-score shows no age trend, with medians ranging from 10th to 30th centile, and *ASXL3* is lowest, on the 3rd centile. In 19/24 cases, the centiles for the two sexes are the same on the z-score scale. Our LMSz method produces gene-specific growth charts for rare diseases, which, when used in the correct context, could be an important clinical tool. We plan to automate it within the DECIPHER platform, enabling availability for relevant genes.

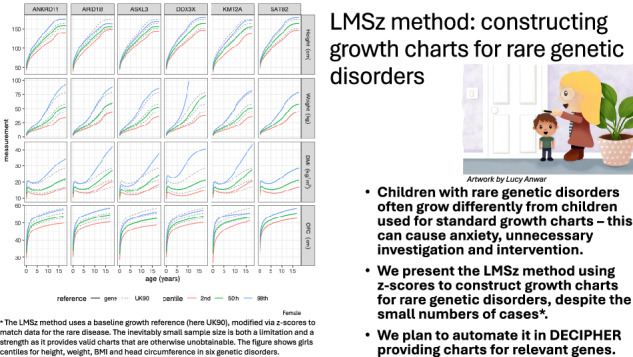

## Introduction

Health professionals measure and plot children’s growth at intervals on appropriate charts to track the trajectory against reference centiles [1]. Many children with genetic disorders plot on an extreme centile—in itself a clue to a possible underlying genetic diagnosis [2]. Once a genetic diagnosis is made, plotting on a standard chart may be misleading. It may, for example, suggest a child is of short stature and underweight when they are growing normally for their genetic disorder, leading to clinical/parental anxiety resulting in unnecessary investigation and unwarranted/ineffective intervention. Conversely, abnormal growth may be incorrectly ascribed to the underlying genetic disorder, and other causes left untreated. Gene-specific growth charts are therefore important in paediatric care. However, due to the small numbers of affected individuals, few gene-specific growth charts are available [3–5].

This proof-of-principle study tests a new method for constructing growth charts in rare disorders based on small datasets, which we call the LMSz method. We call it this because it applies the LMS method [6] on the z-score scale.

## Methods

Growth data were collated from the Deciphering Developmental Disorders (DDD) [7] and GenROC study [8] datasets, both from the UK. For proof-of-principle, we selected *ANKRD11*, a frequently diagnosed gene in the DDD study with a well-described phenotype. We included only participants with pathogenic or likely pathogenic *ANKRD11* variants, excluding any with composite genetic diagnoses. We extracted data on sex, gestational age, birth weight, birth occipitofrontal circumference (OFC), and longitudinal measures of height, weight and OFC and the associated ages at measurement.

For *ANKRD11*, we undertook a second phase of analysis, including European datasets (Spain, Denmark and the Netherlands). We also extended the analysis to five other genes: *ARID1B*, *ASXL3*, *DDX3X*, *KMT2A* and *SATB2*. *ASXL3* growth data were collated from the DDD study and the *ASXL3* international natural history study (IRAS: 316055). We compared our *ARID1B* charts with the Coffin-Siris Syndrome heterogeneous gene group growth charts [4].

We also analysed data from the Mowat Wilson Syndrome (MWS) Growth Chart Consortium and compared our results with those obtained by fitting the raw data using the LMS method (see Supplement 1 for details). They were also compared to the corresponding centile charts published by the MWS Consortium [3].

Age was adjusted for gestation using the formula ${adjusted\; age}={age}+\left({gestation}-40\right)\times 7/365.25$. Body mass index (BMI) was calculated as ${weight}/{{height}}^{2}$ in units of ${{\mathrm{kg}}}/{{{{\rm{m}}}}}^{2}$.

### Statistical analysis

Growth centile charts are conventionally constructed using the LMS (lambda-mu-sigma) method [6], a special case of the family of Generalised Additive Models for Location, Scale and Shape (GAMLSS) [9], where LMS corresponds to the Box-Cox Cole Green or BCCG family. Using anthropometry from a reference sample of children, the LMS method estimates three smooth curves in age. M is the median or 50th centile curve; the S curve is the coefficient of variation or fractional standard deviation (SD), and the L curve is a measure of skewness. Any required centile curve can be constructed from the LMS curves, and by reversing the process, data for individuals can be converted to exact z-scores. The z-score conversion works as follows: the measurement is first expressed as a centile of the underlying BCCG distribution, then the centile is converted to its Normal equivalent deviate or z-score. The conversion from z-score to measurement reverses the process. In this way, the more complex BCCG distribution is mapped to a Normal distribution.

The LMS method is most effective with large datasets, ideally many thousands of individuals [10]. The issue with charts for rare diseases is the inherent scarcity of data. In this case, the chart cannot be based solely on the available data; it must “borrow strength” from previous knowledge about how children grow, as contained in an existing or “baseline” growth reference. This baseline reference can then be adjusted using the rare disease data to obtain a reference for the rare disease. This is analogous to a Bayesian analysis where prior information (the baseline reference) is combined with rare disease data to give a posterior or updated reference relevant to the rare disease.

The process is as follows: the data are first converted to z-scores using the baseline reference, which here is the British 1990 reference (UK90) [11, 12], the official UK chart for birth and age 4–20, and the reference used by the DECIPHER database. Switching to the z-score scale creates centile curves, which are horizontal straight lines, as each centile curve connects points with the same z-score; these z-score “centiles” are also the same for both sexes. For representative children, the z-transformed centiles are Normally distributed with mean (and median) 0 and SD 1 at all ages. Both mean and SD can then be viewed as straight lines plotted against age. Other centile curves are lines above or below the median, spaced according to the corresponding z-score (e.g. the 97.7th centile is at *z* = 2, i.e., 2 SDs above the mean).

The LMSz method now makes four strong though plausible assumptions. It assumes that (1) the rare disease median curve is also a straight line, but estimated from the rare disease data as the linear regression of UK90 z-score on age, and that (2) this line may be different for the two sexes. The SD curve is also assumed (3) linear and estimated in the same way from the linear regression of the z-score SD on age and sex. GAMLSS is used to estimate the mean and SD regression lines simultaneously, using the Normal or NO distribution family. The two lines are analogous to the M and S curves of the LMS method, but on the z-score scale, and are called the M line and S line. Note that there is no equivalent L line as the normal distribution is by definition not skew, and it is also assumed that (4) conversion from measurement to z-score removes any skewness.

The GAMLSS regression equation for the M line is made up of four terms: intercept, age trend, mean sex difference and age by sex interaction (i.e., the age trend differing between the sexes), and the coefficient for each term is estimated:

$$M={a}_{M}+{b}_{M}\times {age}+{c}_{M}\times {sex}+{d}_{M}\times {age}\times {sex}$$

But if one or more of the fitted coefficients is small enough, the corresponding term(s) can be dropped from the regression, leading to a set of five progressively simpler equations:

$$M={a}_{M}+{b}_{M}\times {age}+{c}_{M}\times {sex}$$

$$M={a}_{M}+{b}_{M}\times {age}$$

$$M={a}_{M}+{c}_{M}\times {sex}$$

$$M={a}_{M}$$

$$M=0$$

The SD or S line is modelled in the same way, except it is log-transformed to ensure positivity. The interaction term is also omitted for simplicity.

$${log} (S)={a}_{S}+{b}_{S}\times {age}+{c}_{S}\times {sex}$$

$$log (S)={a}_{S}+{b}_{S}\times {age}$$

$$log (S)={a}_{S}+{c}_{S}\times {sex}$$

$$log (S)={a}_{S}$$

$$log (S)=0$$

Note that the final equations in these lists are null models where the coefficient is constrained to be 0. They correspond to M = 0 and S = 1, the mean and SD for the baseline UK90 reference, which are also the default values for the GAMLSS NO distribution. This means that in cases where the best-fitting model is the null model, it is equivalent to using UK90 as the gene-specific reference. GAMLSS estimates the M line and S line simultaneously, and there are six M line equations and five S line equations, so there are $6\times 5=30$ possible model combinations. All 30 are fitted to the data in turn, and they are compared for goodness of fit using either the Akaike information criterion (AIC) or the Bayesian information criterion (BIC), the optimal model being the one with the lowest AIC or BIC. Note that the S line is linear on the log scale, but back-transformed, it is slightly curved if the ${b}_{S}$ coefficient is non-zero.

Lack of fit is measured by the deviance, or −2 log likelihood. The AIC penalises the deviance by adding two units for each coefficient in the model, while the BIC uses a larger penalty of log *n* units per coefficient, where *n* is the sample size. This penalises more complex models more heavily, so they tend to be simpler. For a model where $n=7$, the AIC and BIC are the same, but for larger *n*, the BIC penalty is larger. For example, the most numerous measurement is weight in *ANKRD11,* where there are 488 points and $\log \left(488\right)\approx 6$, so here the BIC penalty is three times the AIC penalty. For this reason, the BIC models are either the same as or simpler than the AIC models.

For larger samples, the set of models for the M line can be extended to include a cubic P-spline curve in age as an alternative to a straight line. This increases the number of possible fitted M lines from six to nine, and the total of combined M + S models from 30 to 45.

Once the optimal model has been identified, it is used to predict the required centile lines on the UK90 z-score scale. They are then back-transformed to the measurement scale using the UK90 reference, giving a set of centile curves appropriate for the rare disease.

### Error-checking

To detect possible data outliers, the data in the form of UK90 z-scores were centred and scaled to gene-specific z-scores, and z-scores exceeding 3.5 in absolute value were excluded from the analysis. In this way, several data errors were identified and either corrected or excluded.

For simplicity, the analysis treats any repeated measures in the data as independent.

### Specificity

To test the specificity of the method, z-scores of measurements with *ANKRD11* (being the most numerous) were replaced by z-scores randomly sampled from a standard Normal distribution, with mean 0 and SD 1. In this way, simulated z-score data were generated with the age and sex structure of the underlying data, where the optimal model ought to be $M=0$ and $\log (S)=0$, corresponding to the baseline UK90 reference. The model was then fitted repeatedly to newly sampled data, and the optimal model noted each time, 100 times each for height (*n* = 355 points), weight (*n* = 488), BMI (*n* = 343) and OFC (*n* = 231).

### Patient participant involvement (PPI)

We sought PPI views on the growth charts from the outset and throughout the process by consulting with the GenROC study established PPI group (parents of children with rare genetic conditions; one young adult with a genetic condition, and a representative of a charity working to support families with genetic conditions) [8]. Results have subsequently been presented to the group as well as to the GenROC study participants via the study newsletter.

## Results

Table 1 provides counts of the number of subjects and data by gene, country, sex and measurement. The age range was restricted to 0–18 years. Linked ethnicity data were not recorded, but we assume the vast majority of cases were Caucasian based on the sources.

**Table 1:** Counts of the number of subjects and data by gene, country, sex and measurement.

Gene	Source	Individuals		Height		Weight		BMI		OFC		Total
		Female	Male	Female	Male	Female	Male	Female	Male	Female	Male	
ANKRD11	DDD	34	50	31	46	60	95	28	43	36	53	392
ANKRD11	GenROC	5	6	6	11	8	18	5	10	2	6	66
ANKRD11	Spanish	33	35	54	78	58	88	53	76	33	48	488
ANKRD11	Danish	3	6	13	13	13	14	13	13	6	3	88
ANKRD11	Dutch	26	40	45	58	64	70	45	57	20	24	383
ARID1B	DDD	38	34	33	24	70	58	29	22	41	33	310
ASXL3	DDD/NatS^a^	49	70	36	52	83	118	32	48	24	41	434
DDX3X	DDD	48	5	36	4	84	8	34	3	47	6	222
KMT2A	DDD	29	43	21	37	50	76	21	32	22	44	303
KMT2A	GenROC	1	4	3	13	4	18	3	13	1	11	66
SATB2	DDD	12	21	9	15	20	37	7	13	12	24	137
Total		278	314	287	351	514	600	270	330	244	293	2889

^a^Natural History Study (International) and DDD(UK).

### Z-score centile charts and growth charts

**Fig. 1:**
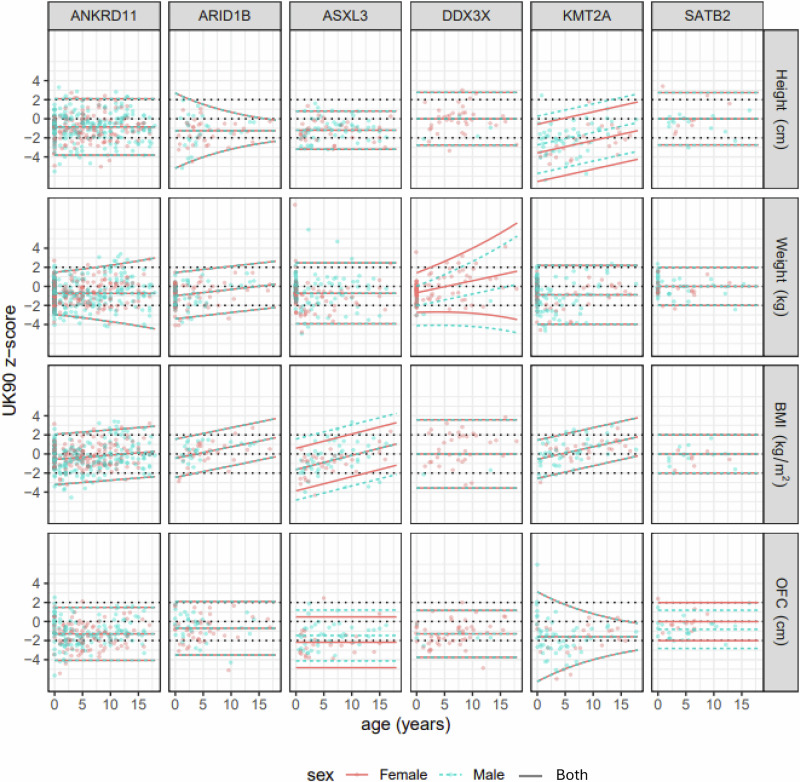
Mean ±2 SD lines representing the 2nd, 50th and 98th gene-specific z-score “centiles” for height, weight, BMI and OFC across the genes *ANKRD11*, *ARID1B*, *ASXL3*, *DDX3X*, *KMT2A* and *SATB2*. The corresponding UK90 z-score centiles at −2, 0 and 2 are shown as dashed lines. Where there is a sex difference, this is shown in red for females and turquoise for males. Where a single grey line is shown, this is due to the centiles being the same for females and males.

The results are presented as a rectangular array of z-score centile charts by gene (*ANKRD11, ARID1B, ASXL3, DDX3X, KMT2A* and *SATB2*) for height, weight, BMI, and OFC (Fig. 1). Three centiles are displayed, corresponding to z-scores −2, 0 and 2, i.e. the 2.3rd, 50th and 97.7th centiles. For simplicity, they are referred to here as the 2nd, 50th and 98th centiles, as is the convention with the UK90 and UK-WHO charts.

In the five plots where there is a sex difference, the centile lines are coloured red for females and turquoise for males. In the plots where the two sets of centiles coincide, they appear as black curves. The raw data are also shown, colour-coded similarly. The *ANKRD11* charts use the data from all four countries, while Supplement 2 discusses the appreciable inter-country differences in *ANKRD11* height.

Figure 1 penalises the plots using the BIC, while Supplementary Fig. 2 shows the same plots penalised with the AIC. As expected, the two sets of centiles are broadly similar, though 13 of the 24 plots in SF2 show a sex difference, as against five in Fig. 1. Similarly, Supplementary Fig. 3 shows the BIC plots of Fig. 1 with the option of a spline curve for the M line. Five of the 24 facets select a spline curve, and in all cases, they show a dip in early life compared to later childhood.

The specificity of the method was assessed with the *ANKRD11* data. Using the BIC models, respectively 98, 97, 97 and 97 times out of 100, the selected model was the null model, i.e., appropriate for the baseline growth reference. This corresponds to an average specificity of just over 97%, i.e., the percentage of times the procedure correctly chooses the baseline reference for data from non-syndromic children. For comparison, repeating the exercise using the AIC models gave a much lower specificity of 53%.

Overall, the differences between Fig. 1 and Supplementary Figs. 2 and 3 are small, and given their lower specificity, there is no obvious reason to prefer the more complicated AIC centiles over the BIC-based centiles, so the focus from here on is the BIC centiles of Fig. 1.

Supplementary Table 1 shows the regression coefficients and standard errors for the models in Fig. 1. Except for weight and BMI for *SATB2*, all the models include one or more significant coefficients, some of them highly so, despite the small sample sizes involved. Conversely, no model includes an age by sex interaction, even though it was tested for. Altogether, there are 53 regression coefficients fitted, which, with 24 distinct gene-measure models, correspond to just over two coefficients per model. Supplementary Table 2 shows the 74 corresponding coefficients for the AIC models.

**Fig. 2:**
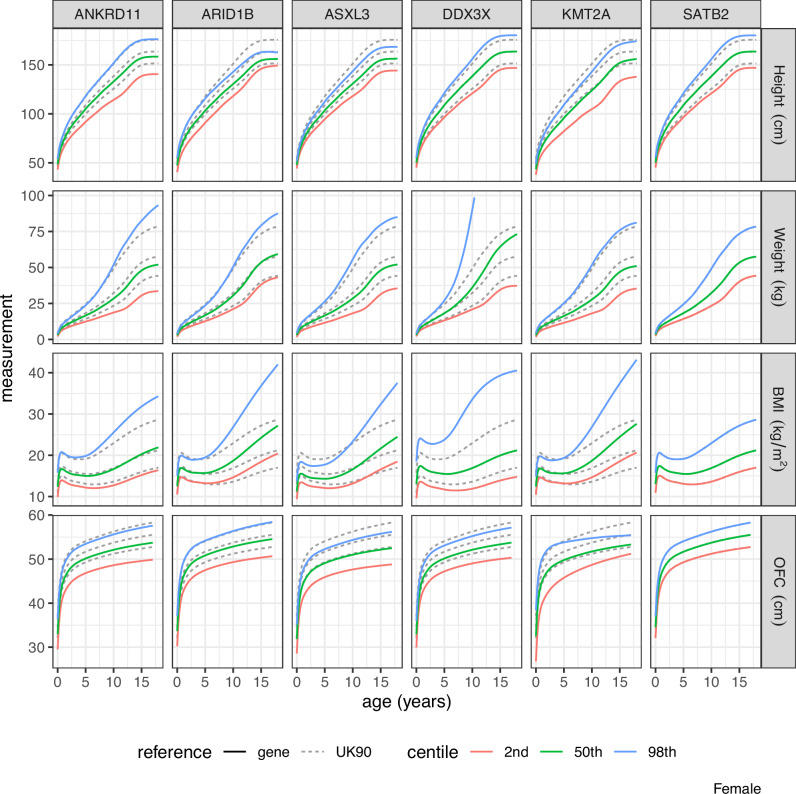
Measurement centiles for females by measurement and gene obtained by back-transforming the z-score centiles shown in Fig. 1. Corresponding UK90 centiles are shown as dashed curves. Where dashed curves are not visible, this is due to the gene centile matching the UK90 centile, such as for SATB2 for weight, BMI and OFC.

**Fig. 3:**
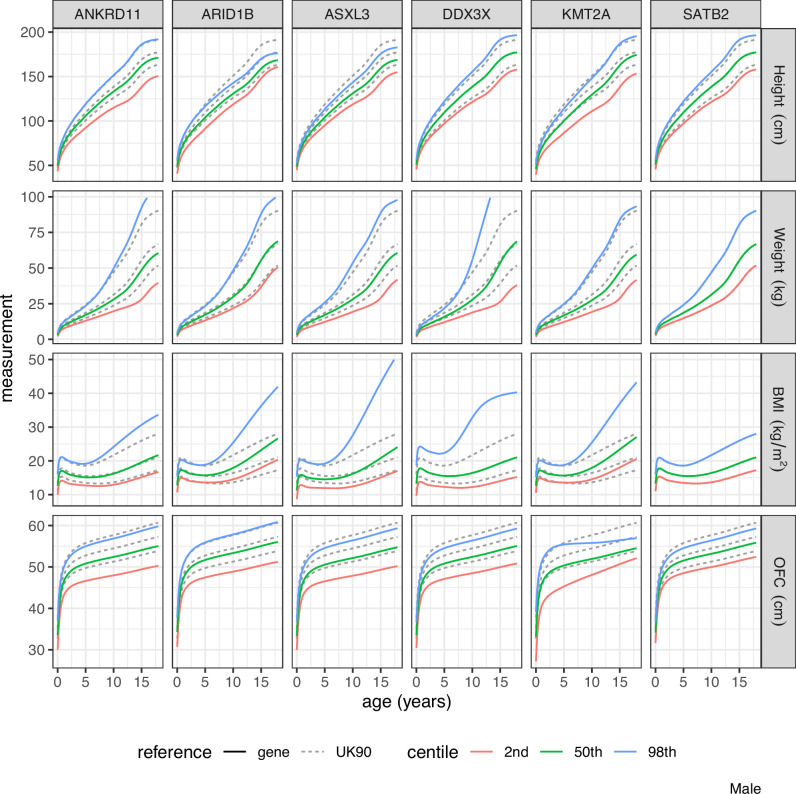
Measurement centiles for males by measurement and gene obtained by back-transforming the z-score centiles shown in Fig. 1. Corresponding UK90 centiles are shown as dashed curves. Where dashed curves are not visible, this is due to the gene centile matching the UK90 centile, such as for SATB2 for weight and BMI.

Figures 2 and 3 show the 2nd, 50th and 98th measurement centiles obtained by back-transforming the z-score centiles of Fig. 1, for females and males, respectively, as a rectangular 4 × 6 plot by measurement and gene, with the corresponding UK90 centiles shown as dashed lines.

### Gene-specific charts

In 19 of the 24 panels in Fig. 1, the z-scores  for males and females are the same, including all four measurements for *ANKRD11*, *ARID1B* and *KMT2A*. The sex differences where they appear are generally small.The fitted model does not include a sex difference which could be due to a lack of data, but more likely it is because the two sexes grow similarly on the z-score scale. Median OFC is constant across age, and except for SATB2 females below the UK90 median, for all the genes. Only *KMT2A* shows a (rising) age trend in median height. But for weight and BMI, two and four genes, respectively, show rising trends, by up to +2 SDs from birth to age 18. This may reflect increasing adiposity with age, as the UK90 reference corresponds to child adiposity as seen in 1990, and obesity prevalence increases with age [13]. In 20 of the panels, the three centiles are parallel lines, indicating that the variability is constant across age. The most striking exception is for weight with *DDX3X*, where the variability is dramatically greater at age 18 than at birth.

Supplementary Table 3 shows the UK90 centiles at birth corresponding to the 2nd, 50th and 98th centiles for each measurement, averaged across sex, by gene.

### 50th centile

The median for *SATB2* (height, weight and BMI) and for *DDX3X* (height and BMI) corresponds to the UK90 median. However, for all other measurements and genes, the median is much lower than the reference, ranging from the 0.08th (height *KMT2A*) to the 28th (BMI *ANKRD11*) UK90 centile.

### 98th centile

The 98th centiles are nearly all above the UK90 90th centile. However, the 98th centile for height in *KMT2A* is on the 44th UK90 centile. Very few *KMT2A* cases achieve even median height on the UK90 chart. Other relatively low 98th centiles are for height in *ASXL3* (79th) and weight in *DDX3X* (76th).

### 2nd centile as z-score

The 2nd centiles at birth are shown as UK90 z-scores because many are far below the 1st UK90 centile. The most extreme cases are *KMT2A* (−6 SD) and *ARID1B* (−5 SD), while all the 2nd centiles are on or below the UK90 2nd centile.

### Mowat Wilson syndrome (MWS) – *ZEB2*

**Fig. 4:**
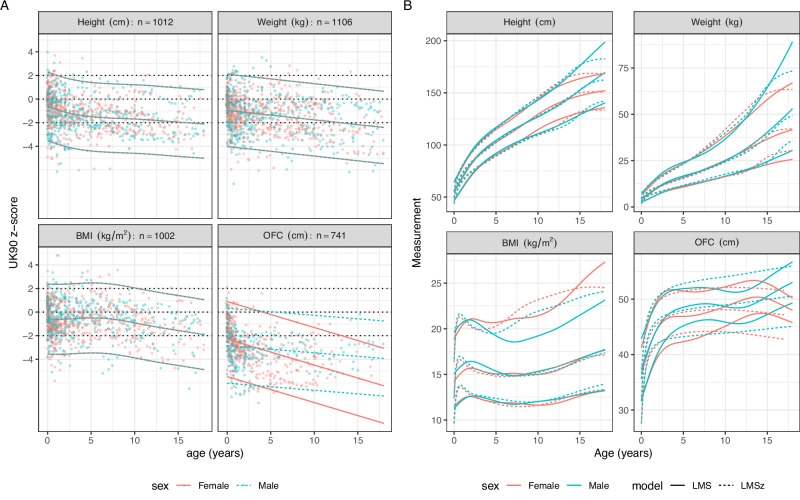
MWS - comparisons of LMS and LMSz using the same dataset. **A** MWS data on the z-score scale, in the same form as Fig. 1. **B** Comparison of the 2nd, 50th and 98th centiles by the two methods, for the sexes separately - solid curves correspond to the LMS method and dashed curves to the LMSz method.

Given the relatively large sample size, we estimated MWS centiles in two ways: the extended version of the LMSz method, including a P-spline M curve as applied to the z-scores (Fig. 4A), and the LMS method as applied to the raw data (Fig. 4B). On the z-score scale (Fig. 4A), the medians for height and BMI are curves, and all four measurement medians are below the UK90 median at birth, with the OFC median close to the UK90 2nd centile. The medians all fall further with increasing age, and at a faster rate for OFC among the females. Figure 4B compares the 2nd, 50th and 98th centiles by the two methods, for the sexes separately, where the solid curves correspond to the LMS method while the dashed curves are for the LMSz method. For height, weight and BMI, there is reasonable agreement, though the BMI 98th centiles agree less well. The height and weight centiles are consistent by sex, being similar until puberty, when the males become taller and heavier. However, unlike the LMSz centiles, the LMS centiles continue rising after puberty despite growth ending, because there are too few data at older ages to pull them down. OFC performs poorly, with all three LMS-based centiles rising and falling at different ages, while the LMSz centiles rise linearly for males but fall in later childhood for females.

## Discussion

We have developed a method for producing gene-specific z-score centile charts and growth charts based on small datasets in rare diseases. The resulting charts are unbiased due to the way they are constructed; cosmetically, they look plausible; they agree with other published literature, and the centiles almost always exactly match the baseline reference when given random z-scores, i.e., the specificity is as high as 97%.

### Sample size

Growth charts typically require many thousands of data points to ensure adequate precision for the outer centiles [10]. This does not work for the small samples available in rare disease cohorts. The LMSz method instead considers the universe of all possible simple models, such as all combinations of sex and linear age for the mean and SD, and selects the best model, penalised for complexity. This works because the universe of all possible linear models is small, between 30 and 45 alternative models, and the model selection does not depend on the statistical significance of the regression coefficients.

### Fitting of centiles

Due to the larger penalty per extra coefficient, the BIC models ought to be simpler than the AIC models, and indeed they are—the BIC models underlying the 24 facets in Fig. 1 involve 53 coefficients, i.e., ~2 coefficients per facet. In contrast, the corresponding AIC models in Supplementary Fig. 2 involve 74 coefficients, averaging over 3 per facet. Despite this, the centiles in Fig. 1 and Supplementary Fig. 2 are not materially different, probably because the coefficients are all small. The specificity calculation showed that the AIC models had only 53% specificity, compared to 97% for the BIC models, for selecting the correct null model when the data were random Normal. This shows that the AIC tends to choose over-complex models, which in turn supports the use of the BIC models. Note that the sensitivity of the method cannot be tested in the same way as the specificity. It involves knowing in advance the centiles appropriate for the particular genotype, which are by definition unknown.

### Z-scores versus growth centiles

The z-score centile plots show clearly the differences in growth pattern for a particular gene compared to UK90. The z-score plots compactly show growth trends over time, and sex differences where present. However, in clinical practice, health professionals measure and plot their patients’ growth on centile charts as part of standard clinical care. For this reason, it is important to provide the back-transformed centile charts for each gene, see Figs. 2 and 3, as clinicians will find them more familiar in measurement units.

### Comparison with the literature and published charts

There is a published centile chart for Coffin-Siris Syndrome (CSS; OMIM #135900), caused by variants in BAF complex genes [4]. Direct comparison with *ARID1B* is not possible given the greater genetic heterogeneity and differing endpoints (height to age 10; OFC to 36 months). In fact, we did not see a sex difference for weight, but the 50th centile for height at age 10, and weight and OFC were consistent.

We were able to analyse the MWS dataset in two ways: LMSz as described here and the conventional LMS method applied to the raw data. The two sets of centiles are reasonably similar, though the z-score-based centiles look more convincing for height and weight, particularly at older ages. For BMI, the 2nd and 50th centiles agree well, but the 98th centiles are more discordant. This is probably because the z-score conversion applies the UK90 skewness adjustment to the MWS data, but the degree of skewness in the MWS data is different from UK90. With right skewness, this would tend to affect the upper rather than the lower centiles of the distribution, as seen here.

MWS centiles have already been published by the MWS Consortium [3], but superficially they look different from the centiles in Fig. 4. This may be because we excluded different outliers and fitted a different model–see Supplement 1 for our gamlss code.

Clinically, the dashed curves are more reliable, particularly at older ages, because the LMSz method preserves the expected pattern of growth (e.g., plateauing after puberty), even when data are sparse, by anchoring to the external reference. In contrast, the LMS method applied directly to raw data can overfit and artificially extend growth trends, as seen where the LMS centiles continue to rise post-puberty despite growth having ended.

There are no published growth charts for *ANKRD11*, *KMT2A*, *DDX3X* or *ASXL3*. Variants in *ANKRD11* cause KBG Syndrome (MIM # 148050) [14]. Our growth charts are consistent with the published phenotypes. Growth hormone has been trialled as a therapeutic option for a small number of individuals with short stature [15]. These growth charts will be an essential tool in monitoring children, both in determining the need for potential treatment and judging its success.

We selected *DDX3X* syndrome (MIM #300160), a neurodevelopmental disorder predominantly in females [16], to investigate how our charts would work for a very rare gene, where $n=5$ for the males. Our charts agree with the literature of fairly normal heights and weights in females, with a proportion having borderline microcephaly. The phenotype in boys is less well understood, as the data are limited. Nonetheless, our method enables some form of chart to be produced. Considered with caution, this could still provide a useful adjunct in a clinical setting, particularly if the data points are viewable in the chart to alert the clinician to the small sample size on which the chart is based.

Alterations in *KMT2A* cause Wiedemann–Steiner Syndrome (WSS) (MIM # 605130) [17]. WSS is associated with short stature in about 60% of individuals, microcephaly in a third, and weight below the 5th centile in a third. Our charts are concordant with published descriptions depicting median height, weight, and OFC for *KMT2A* at the reference 2nd, 2nd and 25th centiles, respectively.

*ASXL3*-related disorder (Bainbridge–Ropers Syndrome, MIM # 615485) is associated with normal birth weight but poor postnatal growth due to feeding issues in infancy, which stabilise following feeding intervention [18–20]. BMI rises with age, which may be explained by sustained feeding intervention or because of dysregulated or impulsive eating behaviours that can develop later in childhood. Feeding interventions should not target reference height (*ASXL3* median height = 12th centile), and our charts will be important for growth monitoring. Median OFC is on the 3rd centile, consistent with the published literature of postnatal microcephaly.

Pre- and postnatal growth restriction, sometimes with microcephaly, can be found in up to 34% of individuals with variants in *SATB2* [21, 22] (MIM # 608148). Growth charts were produced recently (1508 data points), but they are hard to compare with ours as they included single variants together with chromosomal microdeletions, and they stopped at age 10 [5]. They presented z-score heatmaps, which indicate that the microdeletion group was most affected by growth restriction, and this probably explains the difference compared to our charts.

### Limitations

Most individuals were likely Caucasian, although ethnicity was not formally recorded. Data were collated from various sources, and some outliers may be due to measurement error, though we sought out extreme measurements and either corrected or excluded them. Fewer OFC measurements were available, and birth OFC measurements were frequently missing. UK90 was used as the baseline reference for three reasons: most of the data were from the UK (except for the European *ANKRD11* and MWS and International *ASXL3* cohorts), UK90 is the official UK growth reference for data at birth and over 4 years of age [23], and the DECIPHER database uses UK90 to display the growth data. However, there are inter-country differences in height for any child – see Supplement 2 – and this is true for neurodevelopmental disorders, as shown with *ANKRD11 -* clinical interpretation needs to take this into account.

The LMSz method converts raw data to z-scores using the baseline reference and then models the z-scores. It assumes that any skewness in the raw data matches the skewness in the reference, and this may not be the case. If the two skewness patterns differ, it will introduce bias, as seen in Fig. 4B for BMI, where the 98th centiles based on the LMS and LMSz methods are very different, particularly in males. BMI is markedly right-skewed at older ages (i.e., the *L*-value is large and negative), which affects the 98th centile more than the 2nd and 50th centiles.

Another limitation is that for conditions that attenuate the pubertal growth spurt, such as achondroplasia [24] and hypochondroplasia [25], the LMSz method will generate rare disease centiles with a spurious pubertal spurt. Clinicians managing such conditions need to bear this in mind.

### Strengths

The LMSz method appears to provide appropriate growth charts for rare genetic conditions where there are, by definition, only small numbers of cases. It is flexible and automatable, which allows rare disease data to be accumulated over time and the charts to be continually updated.

The method relies on a baseline growth reference from which to borrow strength, and here the baseline is the British 1990 or UK90 reference, which was modelled using the GAMLSS BCCG distribution [6, 9]. However, it should be pointed out that the LMSz method will work with *any* GAMLSS model that can convert measurements to z-scores, including other popular distributions such as the Box-Cox *t* (BCT), Box-Cox Power Exponential (BCPE) [26] or generalised gamma (GG) [27]. In addition, it will work with *any* baseline growth reference, not just UK90, and researchers in other countries may prefer to use their own national references where appropriate.

### Implementation

We plan to automate LMSz within the DECIPHER platform [28, 29] using its open-access datasets alongside further growth measurements being collated through the GenROC study [8]. Gene-specific growth charts will be viewable within DECIPHER. A future development would be to vary subtype-specific charts within our proposed pipeline.

## Conclusions

Children with genetic syndromes often have abnormal growth. LMSz allows clinically useful growth charts to be generated from small datasets. These gene-specific growth charts are essential for accurate clinical management of children with genetic conditions. We plan to automate our method in DECIPHER to enable these charts to be available in the future for all paediatric disorder genes.

## Supplementary information

Supplementary material

## Data Availability

Sequence and variant-level data and phenotypic data for the DDD study are available from the European Genome-phenome Archive (EGA; https://www.ebi.ac.uk/ega/) with study ID EGAS00001000775. Clinically interpreted variants and associated phenotypes from the DDD study and GenROC study are available through DECIPHER (https://www.deciphergenomics.org/).
