# Supplementary material for: The LMSz method - an automatable scalable approach to constructing gene-specific growth charts in rare disorders
Source: Eur J Hum Genet. 2025 Oct 13;34(3):348–56. doi: 10.1038/s41431-025-01947-1 (PMC12963436; doi:10.1038/s41431-025-01947-1)
Supplement: Supplementary file 1 — Supplementary material [file 41431_2025_1947_MOESM1_ESM.pdf]

Supplementary Figure 1

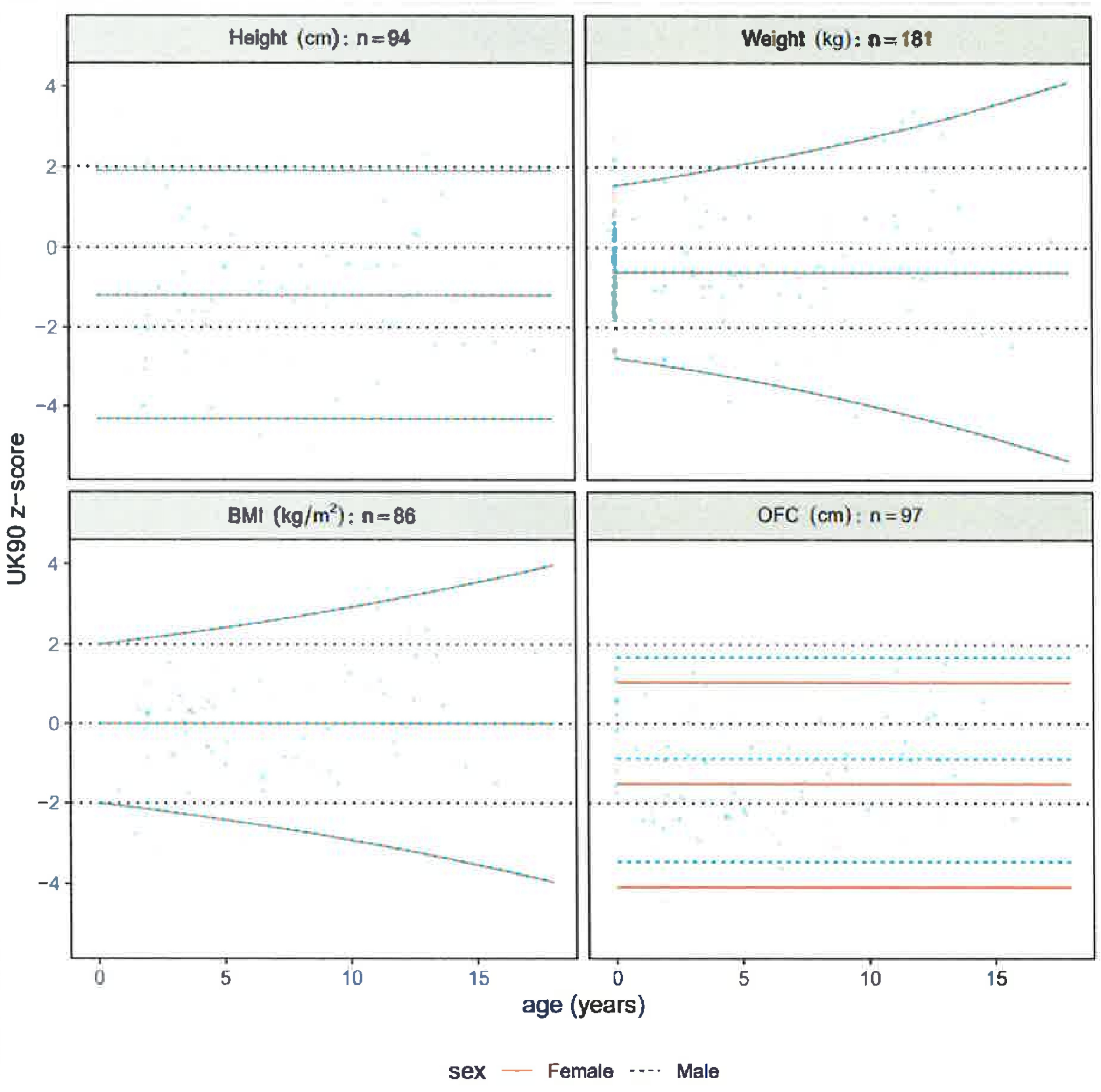

Supplementary Figure 2

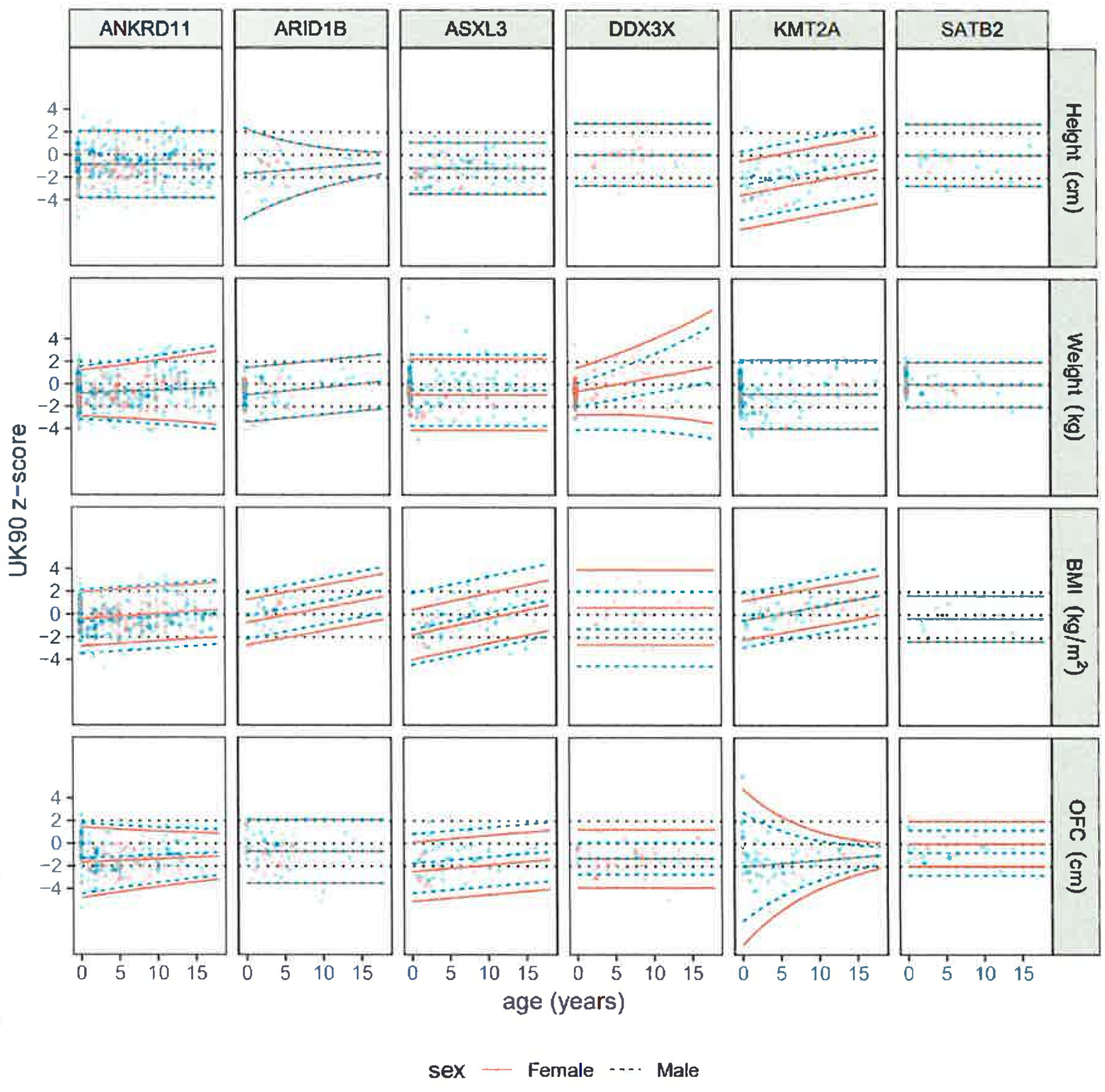

Supplementary Figure 3.

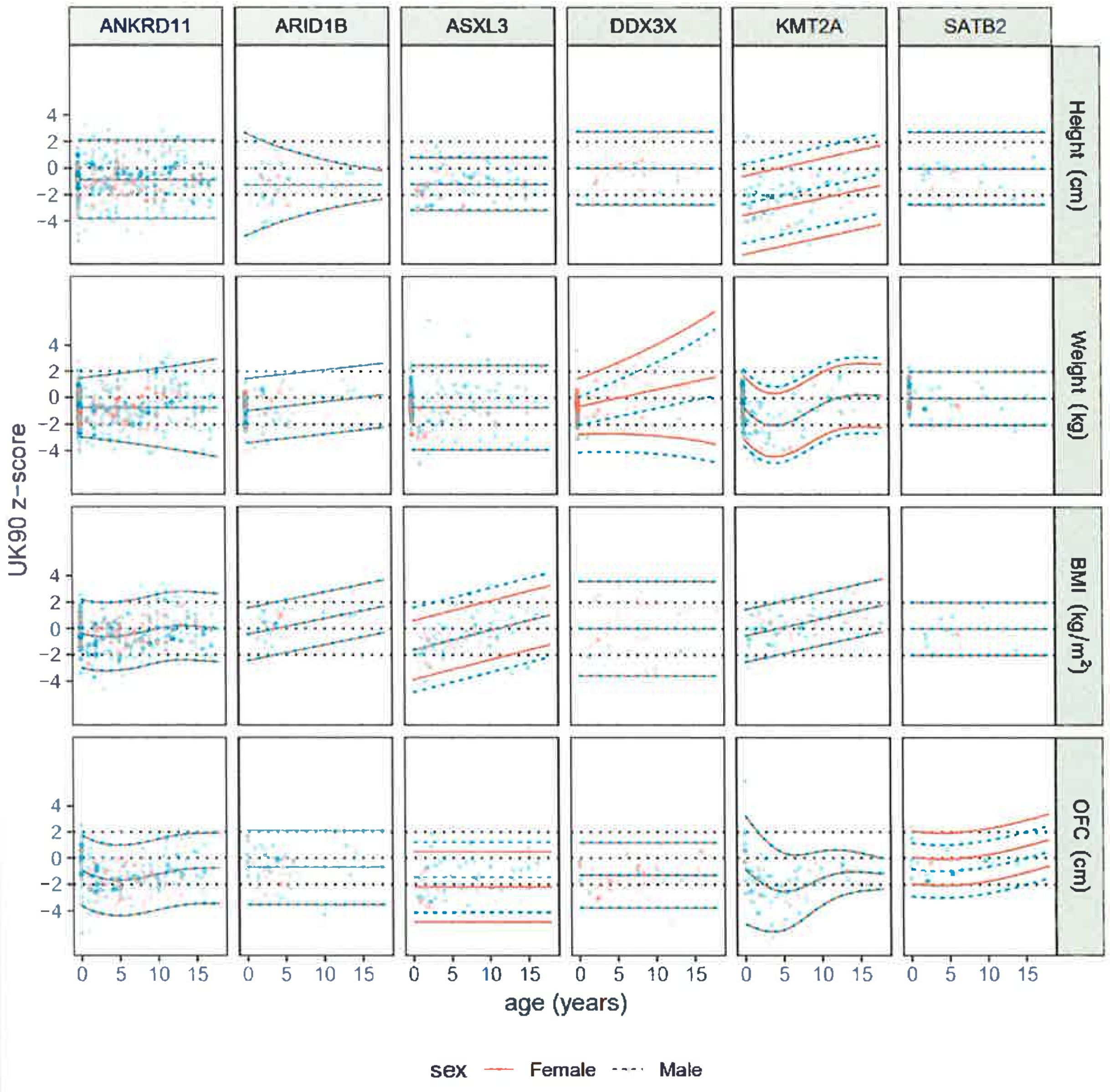

Supplementary Table 1

gene	measure	n	RC_SE	nu.intercep	mu.age	nu.sexMal	gma.interce	sigma.age	gma.sexMale
ANKRD11	Height ~~ (	355	RC	-0.855	0	0	0.389	0	0
ANKRD11	Height ~~ (	355	SE	0.0783	0	0	0.0375	0	0
ANKRD11	Height ~~ (	355	t	-10.9	0	0	10.4	0	0
ANKRD11	Weight ~~	488	RC	-0.742	0	0	0.0997	0.029	0
ANKRD11	Weight ~~	488	SE	0.0561	0	0	0.0428	0.00645	0
ANKRD11	Weight ~~	488	t	-13.2	0	0	2.33	4.49	0
ANKRD11	BMI ~~ (kg	343	RC	-0.585	0.0468	0	0.278	0	0
ANKRD11	BMI ~~ (kg	343	SE	0.111	0.0139	0	0.0382	0	0
ANKRD11	BMI ~~ (kg	343	t	-5.25	3.36	0	7.29	0	0
ANKRD11	OFC ~~ (cn	231	RC	-1.29	0	0	0.329	0	0
ANKRD11	OFC ~~ (cn	231	SE	0.0915	0	0	0.0465	0	0
ANKRD11	OFC ~~ (cn	231	t	-14.2	0	0	7.08	0	0
ARID1B	Height ~~ (	57	RC	-1.25	0	0	0.678	-0.0708	0
ARID1B	Height ~~ (	57	SE	0.172	0	0	0.147	0.0215	0
ARID1B	Height ~~ (	57	t	-7.29	0	0	4.62	-3.29	0
ARID1B	Weight ~~	128	RC	-0.984	0.0665	0	0.193	0	0
ARID1B	Weight ~~	128	SE	0.128	0.027	0	0.0625	0	0
ARID1B	Weight ~~	128	t	-7.71	2.46	0	3.08	0	0
ARID1B	BMI ~~ (kg	51	RC	-0.452	0.12	0	0	0	0
ARID1B	BMI ~~ (kg	51	SE	0.232	0.0349	0	0	0	0
ARID1B	BMI ~~ (kg	51	t	-1.95	3.43	0	0	0	0
ARID1B	OFC ~~ (cn	74	RC	-0.706	0	0	0.338	0	0
ARID1B	OFC ~~ (cn	74	SE	0.163	0	0	0.0822	0	0
ARID1B	OFC ~~ (cn	74	t	-4.33	0	0	4.11	0	0
ASXL3	Height ~~ (	88	RC	-1.2	0	0	0	0	0
ASXL3	Height ~~ (	88	SE	0.107	0	0	0	0	0
ASXL3	Height ~~ (	88	t	-11.2	0	0	0	0	0
ASXL3	Weight ~~	201	RC	-0.727	0	0	0.47	0	0
ASXL3	Weight ~~	201	SE	0.113	0	0	0.0499	0	0
ASXL3	Weight ~~	201	t	-6.45	0	0	9.42	0	0
ASXL3	BMI ~~ (kg	80	RC	-1.64	0.149	0	0.11	0	0.36

ASXL3	BMI ~~ (kg	80	SE	0.259	0.0326	0	0.127	0	0.167
ASXL3	BMI ~~ (kg	80	t	-6.34	4.58	0	0.866	0	2.16
ASXL3	OFC ~~ (cn	65	RC	-2.18	0	0.714	0.287	0	0
ASXL3	OFC ~~ (cn	65	SE	0.272	0	0.342	0.0877	0	0
ASXL3	OFC ~~ (cn	65	t	-8.01	0	2.08	3.27	0	0
DDX3X	Height ~~ (	40	RC	0	0	0	0.321	0	0
DDX3X	Height ~~ (	40	SE	0	0	0	0.112	0	0
DDX3X	Height ~~ (	40	t	0	0	0	2.87	0	0
DDX3X	Weight ~~	92	RC	-0.662	0.125	-1.39	0.0353	0.0497	0
DDX3X	Weight ~~	92	SE	0.144	0.037	0.423	0.0925	0.0169	0
DDX3X	Weight ~~	92	t	-4.6	3.37	-3.27	0.382	2.93	0
DDX3X	BMI ~~ (kg	37	RC	0	0	0	0.581	0	0
DDX3X	BMI ~~ (kg	37	SE	0	0	0	0.116	0	0
DDX3X	BMI ~~ (kg	37	t	0	0	0	5	0	0
DDX3X	OFC ~~ (cn	53	RC	-1.29	0	0	0.208	0	0
DDX3X	OFC ~~ (cn	53	SE	0.169	0	0	0.0971	0	0
DDX3X	OFC ~~ (cn	53	t	-7.62	0	0	2.14	0	0
KMT2A	Height ~~ (	74	RC	-3.58	0.13	0.842	0.407	0	0
KMT2A	Height ~~ (	74	SE	0.456	0.0393	0.395	0.0822	0	0
KMT2A	Height ~~ (	74	t	-7.84	3.3	2.13	4.95	0	0
KMT2A	Weight ~~	148	RC	-0.886	0	0	0.433	0	0
KMT2A	Weight ~~	148	SE	0.127	0	0	0.0581	0	0
KMT2A	Weight ~~	148	t	-6.99	0	0	7.45	0	0
KMT2A	BMI ~~ (kg	69	RC	-0.559	0.131	0	0	0	0
KMT2A	BMI ~~ (kg	69	SE	0.206	0.0255	0	0	0	0
KMT2A	BMI ~~ (kg	69	t	-2.71	5.13	0	0	0	0
KMT2A	OFC ~~ (cn	78	RC	-1.59	0	0	0.856	-0.0669	0
KMT2A	OFC ~~ (cn	78	SE	0.179	0	0	0.122	0.0186	0
KMT2A	OFC ~~ (cn	78	t	-8.92	0	0	7.02	-3.6	0
SATB2	Height ~~ (	24	RC	0	0	0	0.315	0	0
SATB2	Height ~~ (	24	SE	0	0	0	0.144	0	0
SATB2	Height ~~ (	24	t	0	0	0	2.18	0	0
SATB2	Weight ~~	57	RC	0	0	0	0	0	0

SATB2	Weight ~~	57	SE	0	0	0	0	0	0
SATB2	Weight ~~	57	t	0	0	0	0	0	0
SATB2	BMI ~~ (kg	20	RC	0	0	0	0	0	0
SATB2	BMI ~~ (kg	20	SE	0	0	0	0	0	0
SATB2	BMI ~~ (kg	20	t	0	0	0	0	0	0
SATB2	OFC ~~ (cn	36	RC	-0.0109	0	-0.806	0	0	0
SATB2	OFC ~~ (cn	36	SE	0.289	0	0.354	0	0	0
SATB2	OFC ~~ (cn	36	t	-0.0377	0	-2.28	0	0	0

Supplementary Table 2

gene	measure	n	RC_SE	u.intercep	mu.age	nu.sexMal	ma.interce	sigma.age	ma.sexMa
ANKRD11	Height ~~ (	355	RC	-0.855	0	0	0.389	0	0
ANKRD11	Height ~~ (	355	SE	0.0783	0	0	0.0375	0	0
ANKRD11	Height ~~ (	355	t	-10.9	0	0	10.4	0	0
ANKRD11	Weight ~~	488	RC	-0.827	0.0272	0	0.0258	0.026	0.134
ANKRD11	Weight ~~	488	SE	0.068	0.0124	0	0.0557	0.00643	0.0652
ANKRD11	Weight ~~	488	t	-12.2	2.19	0	0.463	4.05	2.05
ANKRD11	BMI ~~ (kg	343	RC	-0.434	0.0466	-0.259	0.174	0	0.162
ANKRD11	BMI ~~ (kg	343	SE	0.128	0.0139	0.14	0.059	0	0.0775
ANKRD11	BMI ~~ (kg	343	t	-3.4	3.36	-1.84	2.95	0	2.09
ANKRD11	OFC ~~ (cn	231	RC	-1.68	0.0306	0.347	0.443	-0.0239	0
ANKRD11	OFC ~~ (cn	231	SE	0.182	0.018	0.179	0.0692	0.00887	0
ANKRD11	OFC ~~ (cn	231	t	-9.2	1.7	1.94	6.4	-2.69	0
ARID1B	Height ~~ (	57	RC	-1.67	0.0513	0	0.705	-0.08	0
ARID1B	Height ~~ (	57	SE	0.314	0.0316	0	0.149	0.0221	0
ARID1B	Height ~~ (	57	t	-5.32	1.62	0	4.73	-3.62	0
ARID1B	Weight ~~	128	RC	-0.984	0.0665	0	0.193	0	0
ARID1B	Weight ~~	128	SE	0.128	0.027	0	0.0625	0	0
ARID1B	Weight ~~	128	t	-7.71	2.46	0	3.08	0	0
ARID1B	BMI ~~ (kg	51	RC	-0.737	0.128	0.56	0	0	0
ARID1B	BMI ~~ (kg	51	SE	0.273	0.0352	0.285	0	0	0
ARID1B	BMI ~~ (kg	51	t	-2.7	3.64	1.97	0	0	0
ARID1B	OFC ~~ (cn	74	RC	-0.706	0	0	0.338	0	0
ARID1B	OFC ~~ (cn	74	SE	0.163	0	0	0.0822	0	0
ARID1B	OFC ~~ (cn	74	t	-4.33	0	0	4.11	0	0
ASXL3	Height ~~ (	88	RC	-1.2	0	0	0.129	0	0
ASXL3	Height ~~ (	88	SE	0.121	0	0	0.0754	0	0
ASXL3	Height ~~ (	88	t	-9.88	0	0	1.71	0	0
ASXL3	Weight ~~	201	RC	-0.949	0	0.377	0.463	0	0
ASXL3	Weight ~~	201	SE	0.174	0	0.228	0.0499	0	0
ASXL3	Weight ~~	201	t	-5.44	0	1.66	9.28	0	0
ASXL3	BMI ~~ (kg	80	RC	-1.81	0.146	0.461	0.0957	0	0.361
ASXL3	BMI ~~ (kg	80	SE	0.275	0.0322	0.3	0.125	0	0.162
ASXL3	BMI ~~ (kg	80	t	-6.59	4.53	1.53	0.764	0	2.23
ASXL3	OFC ~~ (cn	65	RC	-2.51	0.0595	0.702	0.267	0	0
ASXL3	OFC ~~ (cn	65	SE	0.334	0.0365	0.336	0.0877	0	0
ASXL3	OFC ~~ (cn	65	t	-7.5	1.63	2.09	3.04	0	0
DDX3X	Height ~~ (	40	RC	0	0	0	0.321	0	0
DDX3X	Height ~~ (	40	SE	0	0	0	0.112	0	0
DDX3X	Height ~~ (	40	t	0	0	0	2.87	0	0
DDX3X	Weight ~~	92	RC	-0.662	0.125	-1.39	0.0353	0.0497	0
DDX3X	Weight ~~	92	SE	0.144	0.037	0.423	0.0925	0.0169	0
DDX3X	Weight ~~	92	t	-4.6	3.37	-3.27	0.382	2.93	0
DDX3X	BMI ~~ (kg	37	RC	0.618	0	-1.91	0.498	0	0
DDX3X	BMI ~~ (kg	37	SE	0.282	0	0.991	0.116	0	0
DDX3X	BMI ~~ (kg	37	t	2.19	0	-1.93	4.29	0	0
DDX3X	OFC ~~ (cn	53	RC	-1.34	0	0	0.25	0	-0.598
DDX3X	OFC ~~ (cn	53	SE	0.161	0	0	0.103	0	0.313
DDX3X	OFC ~~ (cn	53	t	-8.32	0	0	2.41	0	-1.91

KMT2A	Height ~~ (	74	RC	-3.58	0.13	0.842	0.407	0	0
KMT2A	Height ~~ (	74	SE	0.456	0.0393	0.395	0.0822	0	0
KMT2A	Height ~~ (	74	t	-7.84	3.3	2.13	4.95	0	0
KMT2A	Weight ~~	148	RC	-0.886	0	0	0.433	0	0
KMT2A	Weight ~~	148	SE	0.127	0	0	0.0581	0	0
KMT2A	Weight ~~	148	t	-6.99	0	0	7.45	0	0
KMT2A	BMI ~~ (kg	69	RC	-0.54	0.124	0	-0.15	0	0.327
KMT2A	BMI ~~ (kg	69	SE	0.221	0.0259	0	0.146	0	0.181
KMT2A	BMI ~~ (kg	69	t	-2.45	4.8	0	-1.03	0	1.8
KMT2A	OFC ~~ (cn	78	RC	-2.05	0.0559	0	1.24	-0.101	-0.361
KMT2A	OFC ~~ (cn	78	SE	0.311	0.0277	0	0.232	0.0208	0.207
KMT2A	OFC ~~ (cn	78	t	-6.6	2.02	0	5.35	-4.85	-1.75
SATB2	Height ~~ (	24	RC	0	0	0	0.315	0	0
SATB2	Height ~~ (	24	SE	0	0	0	0.144	0	0
SATB2	Height ~~ (	24	t	0	0	0	2.18	0	0
SATB2	Weight ~~	57	RC	0	0	0	0	0	0
SATB2	Weight ~~	57	SE	0	0	0	0	0	0
SATB2	Weight ~~	57	t	0	0	0	0	0	0
SATB2	BMI ~~ (kg	20	RC	-0.352	0	0	0	0	0
SATB2	BMI ~~ (kg	20	SE	0.224	0	0	0	0	0
SATB2	BMI ~~ (kg	20	t	-1.57	0	0	0	0	0
SATB2	OFC ~~ (cn	36	RC	-0.0109	0	-0.806	0	0	0
SATB2	OFC ~~ (cn	36	SE	0.289	0	0.354	0	0	0
SATB2	OFC ~~ (cn	36	t	-0.0377	0	-2.28	0	0	0

Supplementary Table 3

Measurement	ANKRD11	ARID1B	ASXL3	DDX3X	KMT2A	SATB2
2nd centile (as z-score)						
Height	-3.8	-5.2	-3.2	-2.8	-6.2	-2.7
Weight	-3	-3.4	-3.9	-3.4	-4	-2
BMI	-3.2	-2.5	-4.4	-3.6	-2.6	-2
OFC	-4.1	-3.5	-4.5	-3.8	-6.3	-2.4
50th centile						
Height	20	11	12	50	0.08	50
Weight	23	16	23	8.8	19	50
BMI	28	33	5	50	29	50
OFC	9.8	24	3.4	9.9	5.5	34
98th centile						
Height	98	100	79	100	44	100
Weight	93	93	99	76	99	98
BMI	98	94	86	100	93	98
OFC	93	98	80	88	100	94

Supplement 1. Analysis of Mowat Wilson Syndrome data

We undertook z score scale analysis of respectively 1012, 1106, 999 and 741 measurements for height, weight, BMI and OFC. In addition to the z-score analysis, the MWS raw data were also analysed using the LMS method, due to the relatively large sample size. Prior to analysis, the data were checked for outliers as described in the main text. A total of 37 measurements were excluded, where the internal z-score exceeded 3 in absolute value.

The GAMLSS code to fit the model was as follows:

```
library(gamlss)
control <- pb.control(inter = 10)
model <- gamlss(value ~ pb((age+3/4)^1/4, max.df = 6, control = control),
       sigma.fo = ~pb(age, max.df = 3, control = control),
       data = data, family = BCCGo, nu.start = 1,
       nu.fix = measure %in% c('Height', 'OFC'))
```

Here `value` corresponds to height, weight, BMI or OFC. The median mu curve is fitted as a P-spline in age restricted to 6 or fewer degrees of freedom (d.f.), where age is transformed to over-sample the early measurements – see Cole (2021). The sigma curve is a P-spline with 3 or fewer d.f., and the nu curve is a constant. In addition nu is forced to 1, i.e. a Normal distribution, for height and OFC.

Cole TJ. 2021. Sample size and sample composition for constructing growth reference centiles. Stat Methods Med Res 30:488-507.

Supplement 2. Height differences by country

Figures 1, 2 and 3 show results for height in ANKRD11 based on pooled data from the UK, Spain, Denmark, and the Netherlands. The fitted z-score model consists of intercepts of -0.85 for the mean and 0.39 for the log SD (i.e. SD = 1.5) (see Supplementary Table 1), indicating marked short stature and increased variability compared to the UK90 reference.

To test for the possibility of inter-country differences in mean height, the model was extended to include an adjustment for *country*, and the country mean coefficients are shown in the table, with the overall mean subtracted. Thus the values indicate the country-specific offset to be applied to the centiles from the pooled model in Figure 1. For example centiles for UK children are on average about 0.33 z-scores (half a centile channel width) lower, and Dutch children half a channel width higher, than in Figure 1. These country differences can be taken into account by clinicians when managing their patients.

	UK<br>n = 94	Spain<br>n = 132	Denmark<br>n = 26	Netherlands<br>n = 103
Mean	-0.35	-0.10	0.23	0.38
Std. Error	0.15	0.13	0.28	0.14

So Dutch children are highly significantly taller than UK children, by more than a channel width on the chart, which implies that *country* should be added to the regression model. However the penalty associated with adding the three extra degrees of freedom to the model proved to be larger than the reduction in deviance, so its BIC was larger than the simpler model. Hence even if the list of models used in the LMS-z method had included *country*, it would not have been selected.

Non-syndromic children are also known to be taller in the Netherlands than the UK, and the same may well apply to the other syndrome groups in Figure 1. The centiles for the

non-ANKRD11 genes are based entirely on UK children, so they may be up to a channel width too low for Dutch children, though this has to be speculative given the absence of further Dutch data.
